# Hepato-Immune Crosstalk Underlies Antipsychotic-Associated Metabolic Dysregulation in Schizophrenia: Quercetin as a Therapeutic Candidate for High-Inflammatory Subtypes

**DOI:** 10.3390/metabo16100733

**Published:** 2026-09-29

**Authors:** Yi Yang, Bin Liu, Zhicheng Cao, Haiping Fang, Yi Guo, Tingting Jiang, Ying Lu, Shumin Qu, Qiang Tang, Yingyi Wang, Ying Xia, Xirong Sun, Yiying Sun

**Affiliations:** Shanghai Pudong New Area Mental Health Center (Tongji University Affiliated Mental Health Center), Tongji University Clinical Research Center for Mental Disorders, Shanghai 200124, China

**Keywords:** schizophrenia, antipsychotic drugs, metabolic disorders, liver-immune crosstalk, molecular subtypes, quercetin

## Abstract

**Background**: Patients with schizophrenia (SCZ) receiving antipsychotic treatment frequently develop metabolic complications, yet the mechanisms underlying liver-immune crosstalk remain poorly characterized. **Methods**: This study recruited 340 antipsychotic-treated SCZ patients and 340 matched healthy controls, with a 2-year follow-up restricted to the patient cohort. Candidate genes with hepatic metabolic relevance were screened from the independent GSE21138 brain transcriptomic dataset using machine learning. Molecular docking was performed to predict binding interactions of quercetin as a candidate bioactive compound. **Results**: Long-term antipsychotic exposure was associated with elevated serum 9-hydroxyrisperidone (9-OH-RISP) and substantial changes in liver function and metabolic markers; drug metabolite concentrations correlated with hepatic indices. Transcriptomic analysis identified 317 differentially expressed genes, from which 26 genes with liver-metabolism-related functions were selected as candidates. Seven hub genes (*ABHD6, ALDH4A1*, *ETNPPL*, etc.) were further identified via Support Vector Machine and Random Forest algorithms, and these genes were enriched in metabolic pathways, ferroptosis and other key signaling cascades. Unsupervised clustering based on hub gene expression stratified patients into high-inflammation (C2) and low-inflammation (C1) subtypes. The C2 subtype displayed computationally estimated enrichment of immune cells including B cells and naive CD8^+^ T cells. Molecular docking predicted strong binding affinity (binding energy ≤ −7.0 kcal/mol) between quercetin and proteins encoded by six hub genes, implying potential multi-target anti-inflammatory and metabolic–regulatory interactions. **Conclusions**: This study highlights the involvement of liver–immune crosstalk in antipsychotic-associated metabolic perturbations. The findings suggest exploratory potential for quercetin as a candidate bioactive compound in high-inflammatory SCZ subtypes, though these in silico predictions require further experimental validation.

## 1. Introduction

Schizophrenia (SCZ) is a severe chronic mental disorder with a global lifetime prevalence of approximately 0.3% to 0.7%, posing a substantial socioeconomic burden on patients, families, and society [1]. Although antipsychotic medications are the primary treatment for SCZ, second-generation antipsychotics (SGAs), particularly clozapine and olanzapine, are often associated with significant metabolic side effects, including weight gain, hyperglycemia, dyslipidemia, and insulin resistance, collectively known as metabolic syndrome (MetS) [2,3]. Studies have shown that the prevalence of MetS in patients with SCZ can be as high as 32.5% [4], representing a major contributor to elevated cardiovascular disease risk and shortened life expectancy in this patient population. Accordingly, elucidating the understanding of the potential mechanisms of antipsychotic-associated metabolic disorders and the identification of effective intervention strategies are crucial for improving the overall prognosis of patients with SCZ.

As the core organ governing human metabolism, the liver is essential for drug biotransformation and the maintenance of systemic energy homeostasis. Antipsychotic drugs are primarily metabolized in the liver via the cytochrome P450 enzyme system, and individual differences in their metabolism directly affect drug efficacy and the occurrence of adverse reactions [5]. Increasing evidence suggests that both SCZ itself and the long-term use of antipsychotic drugs may induce subtle changes in liver function and remodeling of metabolic pathways [6,7,8]. Nevertheless, the specific molecular mechanisms by which antipsychotic agents systematically disrupt hepatic metabolic networks and subsequently trigger systemic metabolic dysfunction remain poorly elucidated.

In recent years, the role of immune-inflammatory mechanisms in the pathophysiology of SCZ has attracted considerable attention, and the “immune hypothesis” has emerged as a significant theory explaining its pathogenesis [9]. Studies have found that patients with SCZ exhibit phenomena such as complement system activation, cytokine network imbalance, and altered proportions of immune cell subsets in their peripheral blood [10]. The liver, as a vital immune organ, is closely involved in “hepato-immune crosstalk” with the immune system. The occurrence of drug-induced liver injury is strongly associated with abnormal activation of the immune system [11]. Therefore, we hypothesize that antipsychotic drugs may disrupt hepato-immune crosstalk, triggering local and systemic inflammatory responses in the liver, thereby exacerbating metabolic disorders. However, how this crosstalk specifically functions in antipsychotic drug-induced metabolic abnormalities, and whether there are subtype differences among different patients based on immune characteristics, remain to be systematically elucidated.

Against this backdrop, this study aimed to explore the role of liver–immune crosstalk in antipsychotic-associated metabolic abnormalities in SCZ patients. Specifically, we sought to: (1) identify key hepatic metabolic genes related to SCZ metabolic disturbances; (2) characterize molecular subtypes and their distinct immune micro-environment features; and (3) screen potential natural compounds targeting core regulatory targets. This study provides novel insights into the mechanisms underlying SCZ metabolic comorbidities and supports individualized intervention development.

## 2. Methods

### 2.1. Study Participants

This study employed a prospective cohort design. The study participants were patients with mental disorders who participated in free health examinations at the Mental Health Center affiliated with Tongji University between October 2023 and December 2025. Inclusion criteria included: (1) a confirmed diagnosis of SCZ (ICD-10, F20); (2) receiving free medication under community management, including oral antipsychotics (free medication program) or long-acting injectable antipsychotics; (3) voluntarily participating in and completing at least two health examinations. Exclusion criteria included: (1) unclear diagnosis or not meeting the criteria for the study disease; (2) treatment regimen not meeting the requirements of the study groups: not continuously receiving free oral antipsychotics or long-acting injections; (3) incomplete follow-up data: failure to complete baseline (October 2023) or endpoint (December 2025) examinations, or missing core indicators (such as serum drug concentration, metabolic parameters) at two key time points; (4) co-existing severe physical diseases or confounding factors: including but not limited to end-stage liver or kidney failure, malignant tumors, uncontrolled thyroid dysfunction, or long-term use of concomitant medications that may significantly affect metabolism or drug concentration; (5) pregnant or lactating women; (6) participation in other interventional clinical trials that may interfere with the observed indicators of this study.

This longitudinal study enrolled 340 patients with SCZ (ICD-10, F20) who met the inclusion and exclusion criteria. To ensure diagnostic consistency, patients with other psychotic disorders were strictly excluded. All subjects underwent standardized physical examinations and laboratory tests every six months. Clinical data were collected continuously from October 2023 (baseline) to December 2025 (two-year follow-up endpoint) to investigate longitudinal changes in hematological parameters, hepatic and renal function, and glucose and lipid metabolism markers during stable antipsychotic treatment. Prior to enrollment, all patients had been receiving long-term treatment with chlorpromazine and/or risperidone; no medication changes, dosage adjustments, additions, or discontinuations occurred throughout the two-year follow-up period. Serum concentrations of chlorpromazine and 9-hydroxyrisperidone (9-OH-RISP) were measured based on each patient’s specific medication regimen; as not all patients were taking both antipsychotics simultaneously, this approach avoided misinterpretations that might arise from treating the entire cohort as receiving uniform combination therapy.

Sample size justification and primary endpoint definition were clarified for this longitudinal cohort design. The present study adopted serum 9-OH-RISP as the primary research endpoint, which is the major pharmacologically active metabolite of risperidone. It stably reflects in vivo long-term antipsychotic exposure and drug metabolic activity, and is closely associated with hepatic and metabolic phenotypic alterations, thus serving as a reliable biomarker for evaluating treatment-related biochemical changes. The sample size calculation was performed based on the longitudinal within-subject design for SCZ patients. With a two-sided α of 0.05 and a statistical power of 0.80, the required minimum sample size was estimated according to previously reported clinical effect sizes of 9-OH-RISP-related metabolic changes [12,13]. Considering potential participant dropout during the two-year follow-up, we ultimately enrolled 340 SCZ patients to ensure sufficient statistical robustness for longitudinal association analysis.

To further elucidate the inherent differences in peripheral blood and liver function markers between patients with SCZ and the general population, 340 age- and sex-matched healthy controls were recruited. None of the healthy controls had psychiatric disorders, chronic systemic diseases, or abnormalities in hepatic or renal function. A cross-sectional comparative analysis of complete blood counts and liver function markers was conducted between the SCZ cohort and the healthy controls at baseline. Notably, only the SCZ cohort underwent longitudinal repeated measurements over the two-year period, whereas the healthy control group underwent only baseline cross-sectional testing without long-term follow-up. This unilateral longitudinal design limited the application of group-by-time interaction analysis using repeated-measures ANOVA or full linear mixed-effects models across two groups; therefore, longitudinal analyses focused on within-patient temporal changes in the SCZ cohort.

### 2.2. Study Variables and Sample Size Calculation

The sample size was estimated based on the inter-group difference in the primary outcome measure-serum 9-hydroxyrisperidone (9-OH-RIS) concentration. According to previous therapeutic drug monitoring studies of risperidone [12,13], the mean plasma concentration of 9-OH-RIS was approximately 31.24 ng/mL, with a standard deviation of approximately 16.07 ng/mL. The minimum clinically significant difference was set at 10 ng/mL. Under the assumption of a two-sided α = 0.05 and 80% power, the formula for comparing two independent samples of continuous variables estimated that approximately 41 cases were needed per group. Considering a 15% dropout rate, 48 cases were needed per group, resulting in a total sample size of approximately 96 cases. The sample size calculation was performed using PASS statistical software (2021 version) before the study. This study ultimately included 340 patients (171 males, 169 females; age 51.57 ± 11.235), with a total sample size of 680 cases. This far exceeded the required sample size, ensuring sufficient statistical power. This study was reviewed and approved by the Ethics Committee of the Mental Health Center affiliated with Tongji University.

$$n_{\mathrm{per}\mathrm{group}}=\frac{2(z_{1-\alpha /2}+z_{1-\beta }{)}^{2}\sigma^{2}}{\Delta^{2}}$$

### 2.3. Laboratory Testing Methods

Venous blood samples were collected from all subjects after at least 8 h of fasting the night before each follow-up visit (baseline: October 2023; follow-up points: April 2024, October 2024, April 2025, and October 2025). Blood samples were sent to the Clinical Laboratory Department of the Shanghai Tongji University Affiliated Mental Health Center for unified testing within 2 h of collection. This laboratory is accredited by the China National Accreditation Service for Conformity Assessment (CNAS), and all testing items were performed according to standardized operating procedures. Complete blood count (five-part differential) was performed using a fully automated hematology analyzer (Sysmex XN-9000, Sysmex Corporation, Kobe, Japan), which provides five-part leukocyte differential counts. Fasting blood glucose was measured using the hexokinase method on a Beckman AU5800 automated biochemical analyzer (Beckman Coulter, Brea, CA, USA). Renal function (excluding creatinine) and electrolytes (sodium, potassium, chloride, calcium) were measured using the ion-selective electrode (ISE) method. The four lipid parameters included total cholesterol (TC), triglycerides (TG), high-density lipoprotein (HDL), and low-density lipoprotein (LDL), all measured using enzymatic methods; LDL-C was calculated using the Friedewald formula when triglycerides were <4.52 mmol/L (400 mg/dL). Liver function tests included: serum albumin, total protein, prealbumin, total bilirubin, direct bilirubin, total bile acids, alkaline phosphatase (ALP), gamma-glutamyl transferase (GGT), alanine aminotransferase (ALT), aspartate aminotransferase (AST), glutathione reductase (GR), and serum fibronectin. These indicators were measured using colorimetric, immunoturbidimetric, or continuous monitoring methods on the Beckman AU5800 automated biochemical analyzer (Beckman Coulter, Brea, CA, USA). All reagents were calibrated by the manufacturer and traceable to national standard reference materials. The plasma concentrations of antipsychotic drugs (chlorpromazine, risperidone, and its active metabolite 9-OH-RISP, etc.) were quantitatively analyzed using ultra-high performance liquid chromatography–tandem mass spectrometry (UHPLC-MS/MS). This method has been validated according to the “Technical Guidelines for Non-clinical Pharmacokinetic Studies of Chemical Drugs” and the FDA bioanalytical method validation guidelines. The lower limits of quantification (LLOQ) for chlorpromazine, risperidone, and 9-OH-RISP were 1.0 ng/mL, 0.5 ng/mL, and 0.5 ng/mL, respectively. The total active drug concentration of risperidone was defined as the sum of the concentrations of risperidone and its main active metabolite, 9-OH-RISP. Each batch of samples included quality control samples at low, medium, and high concentration levels, with intra-batch and inter-batch accuracy deviations < 15% and precision (relative standard deviation) < 15%.

All laboratory personnel involved in the analysis remained blinded to the treatment group and clinical information of the subjects to minimize potential bias.

### 2.4. Data Acquisition and Processing

Transcriptome expression data was obtained from the Gene Expression Omnibus (GEO, https://www.ncbi.nlm.nih.gov/geo/) (accessed on 1 October 2025) database maintained by the National Center for Biotechnology Information (NCBI). GSE21138 is a public transcriptomic dataset containing genome-wide expression profiling of the prefrontal cortex samples from 30 SCZ patients and 29 healthy control individuals. After probe annotation and integration, the expression matrix was further standardized to reduce the impact of technical bias. Subsequently, the “limma” package in R (version 4.4.1) was used to perform statistical analysis of differential gene expression between the healthy control group and the SCZ group. Genes with a *p*-value < 0.05 and a fold change (FC) ≥ 1.3 were considered differentially expressed genes (DEGs).

### 2.5. Screening and Functional Enrichment Analysis of Cross-Tissue Metabolic and Inflammatory Candidate Genes in SCZ

To systematically screen candidate genes with cross-tissue metabolic relevance in SCZ, this study intersected DEGs with liver metabolism-related gene sets to perform exploratory analysis. The liver metabolism-related gene set was obtained from the HPA database (https://www.proteinatlas.org/) [14]. The intersection of these two gene sets yielded preliminary candidate signature genes. To further optimize the signature gene combination and identify key hub genes, we used two machine learning algorithms, Support Vector Machine (SVM) and Random Forest (RF), to rank the importance of the candidate genes. SVM analysis was implemented using the R package e1071 with a linear kernel function; random forest analysis was performed using the R package randomForest, setting the number of decision trees to 1000, and using Mean Decrease Accuracy as the variable importance evaluation metric. Subsequently, the top 10 genes from each algorithm were intersected to finally determine the hub genes for further analysis. To evaluate the diagnostic efficacy of these hub genes in distinguishing SCZ patients from healthy controls, we used Receiver Operating Characteristic (ROC) curves and calculated the Area Under the Curve (AUC). All ROC analyses were performed in the R environment using the pROC package. Furthermore, to elucidate the potential biological functions and signaling pathway associations of the hub genes, we performed pathway enrichment analysis using the Metascape online platform (https://metascape.org). The enrichment results primarily focused on KEGG pathways, with a significance threshold set at *p* < 0.05, and Benjamini–Hochberg correction was applied for multiple testing.

### 2.6. Immune Cell Infiltration Assessment and SCZ Subtype Analysis Based on Hub Genes

This study first used the xCell algorithm to quantitatively assess the immune and stromal cell components of each sample in the GSE21138 dataset. xCell is a deconvolution method based on the principle of gene set enrichment analysis (GSEA), which can simultaneously infer the relative enrichment scores of 64 immune and stromal cell types, including various lymphocytes, myeloid cells, fibroblasts, and an overall stroma score. The raw expression matrix was log2-transformed before input, and the output results were corrected to eliminate the interference of correlations between cell types. Subsequently, to explore potential molecular subtypes of SCZ, we performed unsupervised consensus clustering analysis using the ConsensusClusterPlus R package (version 1.62.0) 9based on the expression profiles of the seven hub genes identified earlier. The clustering parameters were set as follows: resampling iterations were 1000 times, with 80% of the samples drawn each time; Euclidean distance was used as the distance metric, and hierarchical clustering was selected as the clustering algorithm; the exploration range for the number of clusters K was set from 2 to 9. The optimal number of clusters was determined by evaluating the stability of the cumulative distribution function (CDF) curve, the clarity of the consensus matrix blocks, and the relative change rate (Δ(K)). To further compare the differences in the immune microenvironment between different subtypes, we performed inter-group comparison analysis of the xCell immune cell scores of the clustered groups. The Wilcoxon rank-sum test was used for inter-group differences, with a significance threshold of *p* < 0.05. Biological names were assigned to the subtypes based on the overall enrichment level of immune cells.

### 2.7. Screening of Targeted Intervention Agents and Molecular Docking Analysis

To explore potential natural compound intervention strategies, this study used the Enrichr online platform (https://maayanlab.cloud/Enrichr/) (accessed on 1 October 2025) to perform drug–target association analysis based on the seven hub genes identified earlier. This platform integrates multiple pharmacological databases, including DrugBank, DGIdb, and KEGG DRUG, and can predict potential targeted small molecules or natural compounds based on the input gene list. We extracted enrichment results from drug-related gene set libraries such as “Drug Perturbations from GEO” and “DSigDB”, and ranked candidate interventions based on their combined scores, selecting the top 10 compounds for initial screening. Given the advantages of natural flavonoids, such as low toxicity and multi-target effects, quercetin was prioritized as a candidate intervention for further validation. To further assess the binding potential between quercetin and hub gene-encoded proteins, we performed molecular docking simulations. First, the amino acid sequences of the target proteins were obtained from UniProt (https://www.uniprot.org/). For proteins with existing crystal structures (ALDH4A1: 8RKR; ETNPPL: 6TOR; NSUN6: 5WWQ), the three-dimensional structures were downloaded directly from the Protein Data Bank (PDB). For proteins without experimental structures (ABHD6, IQGAP2, SLC39A14), high-confidence predicted models (AF-Q9BV23, AF-Q13576, AF-Q15043) were obtained from the AlphaFold Protein Structure Database (https://alphafold.ebi.ac.uk/) (accessed on 1 October 2025). The three-dimensional structure of quercetin was downloaded from PubChem (CID: 5280343) and subjected to energy minimization and protonation using the Open Babel tool. Molecular docking was performed using AutoDock Vina (version 1.2.0) software. Before docking, water molecules were removed from the receptor protein, polar hydrogens were added, and partial charges were calculated. After flexible optimization of the ligand conformation, a global search was performed within a docking grid box defined around the active site (or the entire protein surface). Each docking run generated 20 conformations, and the one with the lowest binding energy (kcal/mol) was selected as the optimal binding mode. A binding energy ≤ −5.0 kcal/mol was considered the standard for significant binding affinity, based on which the interaction strength between quercetin and each target was determined. Meanwhile, PyMOL (version 2.6) and Discovery Studio (version 2023) were used to visualize and analyze non-covalent interactions such as hydrogen bonds, π–π stacking, and hydrophobic interactions at the key binding interface.

### 2.8. Statistical Analysis

All statistical analyses were performed using SPSS 21.0 and R software (v4.3.1). Continuous variables, including hematological, hepatic, renal, and glucose/lipid metabolism markers, were presented as mean ± standard deviation (SD) for normally distributed data or median (interquartile range, IQR) for non-normally distributed data. Cross-sectional baseline comparisons between patients with schizophrenia (SCZ) and healthy controls were conducted using independent-samples *t*-tests or Mann–Whitney U tests, as appropriate.

Notably, healthy controls only received baseline detection without longitudinal follow-up data at the 2-year time point. Given the absence of longitudinal repeated measurements in the control group, repeated-measures analysis of variance and linear mixed-effects models, which require complete longitudinal data for both groups to evaluate group-by-time interactive effects, were not applicable in the present study. Therefore, paired *t*-tests were only adopted to analyze the longitudinal changes in clinical and biochemical markers within the SCZ patient cohort by comparing baseline and 2-year follow-up data.

Multiple linear regression models were constructed with age and sex as covariates to eliminate the confounding effects of demographic factors on baseline between-group differences in biochemical and hematological parameters. Stratified univariate general linear models (GLMs) adjusted for age and sex were applied to assess subgroup-specific associations between serum antipsychotic (risperidone and chlorpromazine) concentrations and hepatic biomarkers. All analyses were performed separately for risperidone-treated and chlorpromazine-treated patients, and correlation patterns across different medication subgroups were further compared.

Considering the multiple clinical markers included in the study, the Bonferroni correction was used to control for type I errors induced by multiple testing. All statistical tests were two-sided, and a corrected *p* value < 0.05 was defined as the threshold of statistical significance. For transcriptomic bioinformatics analysis, the limma package was utilized for differential expression analysis, and machine learning models were established to screen hub genes associated with hepatic immunometabolic dysregulation.

## 3. Results

### 3.1. Comparison of Blood Routine Tests of the Subjects

This study included 680 participants, comprising 340 healthy controls (age: 55.84 ± 12.98) and 340 patients with SCZ (age: 53.70 ± 11.22). The sex composition of the two groups was identical, with 172 males and 168 females. As shown in Table S1, compared with the control group, patients with SCZ had significantly increased White Blood Cell Count (WBC), Neutrophil Count (NEUT), Monocyte Count (MONO), Platelet Count (PLT), Plateletcrit (PCT), and Mean Platelet Volume (MPV), while Eosinophil Count (EOS), Eosinophil Percentage (EOS%), Red Blood Cell Count (RBC), Hemoglobin (HGB), Mean Corpuscular Hemoglobin (MCH), Mean Corpuscular Hemoglobin Concentration (MCHC), Hematocrit (HCT), Red Cell Distribution Width-SD (RDW-SD), and Red Cell Distribution Width-CV (RDW-CV), with statistically significant differences (*p* < 0.05). After a 2-year follow-up, the relevant indicators of patients with SCZ were remeasured. Statistical analysis of the two groups revealed that after 2 years of follow-up, the basophil count, basophil percentage, platelet count, and plateletcrit were significantly decreased; while platelet distribution width (PDW) and mean platelet volume were significantly increased (Table 1).

### 3.2. Comparison of Liver and Kidney Function Indicators of the Study Subjects

As shown in Table S2, compared with the control group, patients with SCZ had significantly increased levels of Albumin (ALB) and significantly decreased levels of UREA, with statistically significant differences (*p* < 0.05). Statistical analysis of the two groups revealed that after 2 years of follow-up, ALT, AST, total bilirubin (TBIL), total bile acid, uric acid (UA), and albumin–globulin ratio were significantly decreased, while direct bilirubin, total protein (TP), albumin (ALB), globulin, and urea (UREA) were significantly increased (Table 2).

### 3.3. Comparison of Blood Lipid and Blood Glucose Levels of the Study Subjects

As shown in Table S3, compared with the control group, patients with SCZ had significantly increased total Cholesterol (CHOL), Triglycerides (TG) and HDL, and the differences were statistically significant (*p* < 0.05). Statistical analysis of the two groups revealed that after 2 years of follow-up, the fasting blood glucose levels of the study subjects were significantly reduced; HDL levels were significantly increased (Table 3).

### 3.4. Comparison of Blood Drug Concentrations in the Study Subjects

Statistical analysis of the two groups revealed that serum chlorpromazine concentrations in the study subjects decreased significantly after 2 years of follow-up; 9-OH-RISP concentrations increased significantly (Table 4).

### 3.5. Multivariate Linear Regression Analysis of Biochemical Indices and Drug Concentrations

After adjustment for age and gender, multiple linear regression models identified baseline intergroup differences in hematological biomarkers between SCZ patients and healthy controls (Table S4). Patients had elevated WBC, NEUT and MONO but reduced EOS% (β = −0.110, *p* = 0.004). They also presented anemic features with lower RBC, HGB, HCT, MCHC, RDW-SD and RDW-CV (all *p* < 0.001). Platelet indices including PLT, PCT and MPV were markedly increased; PCT carried the largest effect size (β = 0.470, *p* < 0.001).

After two years of stable antipsychotic treatment, patients with SCZ exhibited significant changes in drug metabolism closely associated with CPZ exposure. Liver function markers showed decreases in ALT (β = −0.106, *p* < 0.01), AST (β = 0.181, *p* < 0.01), and TBIL (β = −0.187, *p* < 0.01), alongside an increase in DBIL (β = 0.297, *p* < 0.001), suggesting drug-induced biliary dysfunction. TP, ALB, and GLB levels rose significantly, while the A/G ratio decreased (β = −0.121, *p* = 0.001), consistent with a state of chronic inflammation. HDL-C levels increased (β = 0.122, *p* = 0.001), confirming persistent lipid dysregulation alongside elevated baseline total cholesterol. UREA levels rose (β = 0.092, *p* = 0.011) while UA levels dropped sharply (β = −0.242, *p* < 0.001), indicating alterations in renal excretion and catabolism. Basophil counts declined slightly, and PDW increased (β = 0.087, *p* = 0.024), pointing to changes in immune and platelet status. Serum CPZ was negatively correlated with UA (β = −0.333, *p* = 0.005), supporting the hypothesis of CPZ-mediated inhibition of uric acid excretion. Levels of the active risperidone metabolite 9-OH-RISP also increased (β = 0.243, *p* = 0.011), reflecting ongoing drug metabolism in the body (Table 5). In summary, the biochemical changes observed in SCZ patients after two years of follow-up reflect not only the metabolic disturbances inherent to the disease itself but also highlight the profound impact of antipsychotic medications—particularly CPZ—on hepatic biliary excretion, renal uric acid metabolism, and the immune system.

### 3.6. Correlation Analysis of Various Indicators in the Study Subjects

**Figure 1:**
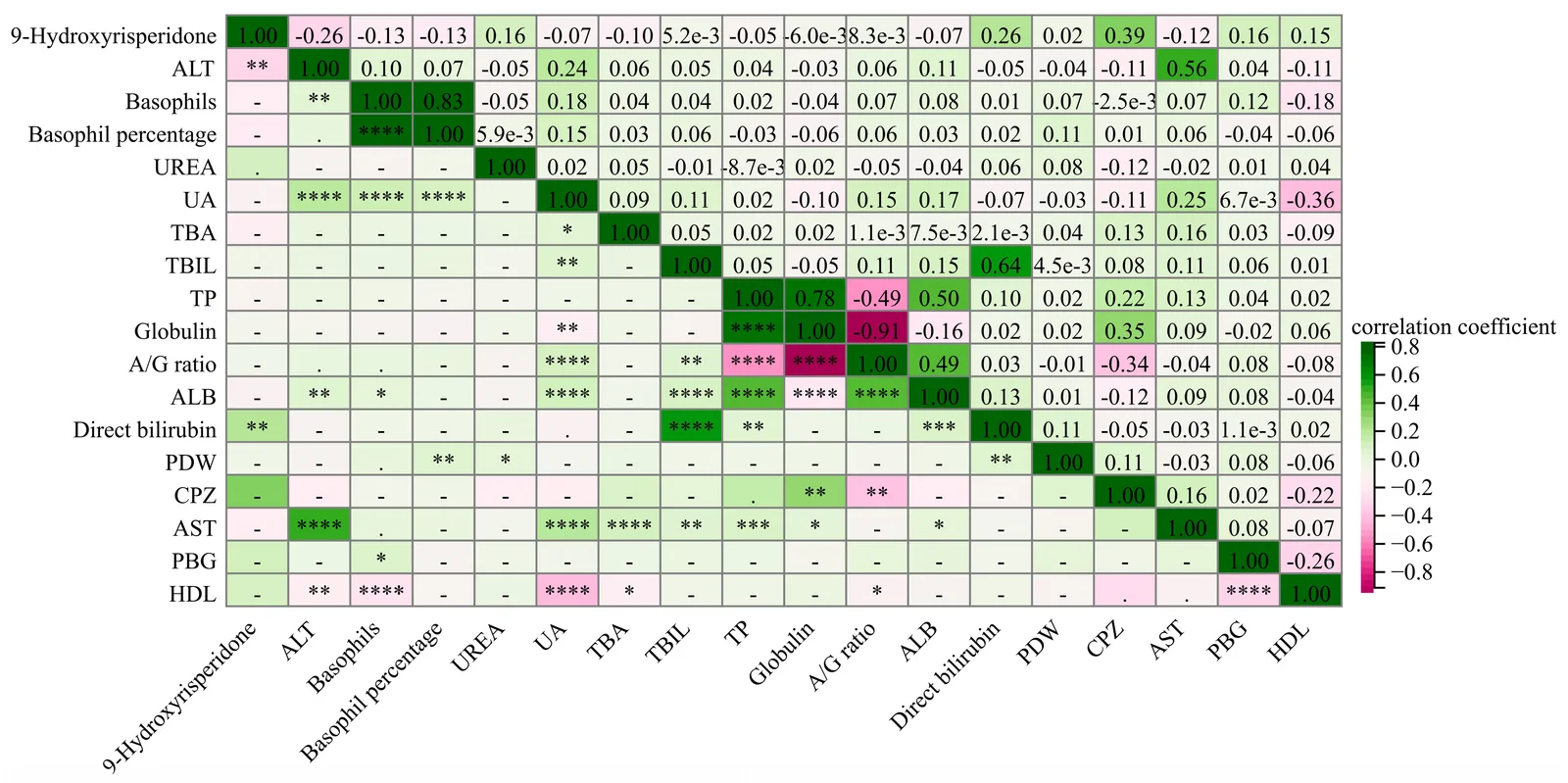
Correlation analysis of various indicators. *—*p* < 0.05; **—*p* < 0.01; ***—*p* < 0.001; ****—*p* < 0.0001.

Correlation analyses (Figure 1) uncovered close organ-specific associations between circulating antipsychotic metabolites and hepatic biomarkers. The active risperidone metabolite 9-OH-RISP was negatively correlated with ALT but positively correlated with DBIL, implying that sustained risperidone metabolism might interfere with hepatocellular enzyme release and biliary excretion function. For CPZ, serum concentration was positively linked to globulin levels and inversely associated with the albumin–globulin ratio, further verifying that cumulative CPZ exposure exacerbates chronic inflammatory immune activation and disrupts hepatic protein homeostasis.

### 3.7. Stratified Regression Analysis of Drug-Specific Hepatic Effects

To further verify the drug-specific independent associations after adjusting for age and sex, stratified univariate general linear models were performed in risperidone-treated and chlorpromazine-treated subgroups separately. Different circulating drug analytes were detected in the two subgroups. In the risperidone subgroup, serum 9-hydroxyrisperidone concentration was significantly correlated with ALT, TBIL, and total protein, while its correlation with AST and direct bilirubin was nearly significant. Conversely, no significant association was found between 9-hydroxyrisperidone and TBA, globulin, A/G ratio, or albumin. In the chlorpromazine subgroup, serum chlorpromazine concentration was significantly positively correlated with AST and globulin, but not significantly correlated with ALT, TBIL, total protein, TBA, albumin, direct bilirubin, or A/G ratio (Table 6).

Overall, these stratified analyses revealed different correlation patterns between drug exposure and changes in liver biochemical parameters, indicating differences in the effects of long-term risperidone and chlorpromazine treatment on liver function and serum protein metabolism.

### 3.8. Screening and Pathway Enrichment Analysis of SCZ-Related Genes

**Figure 2:**
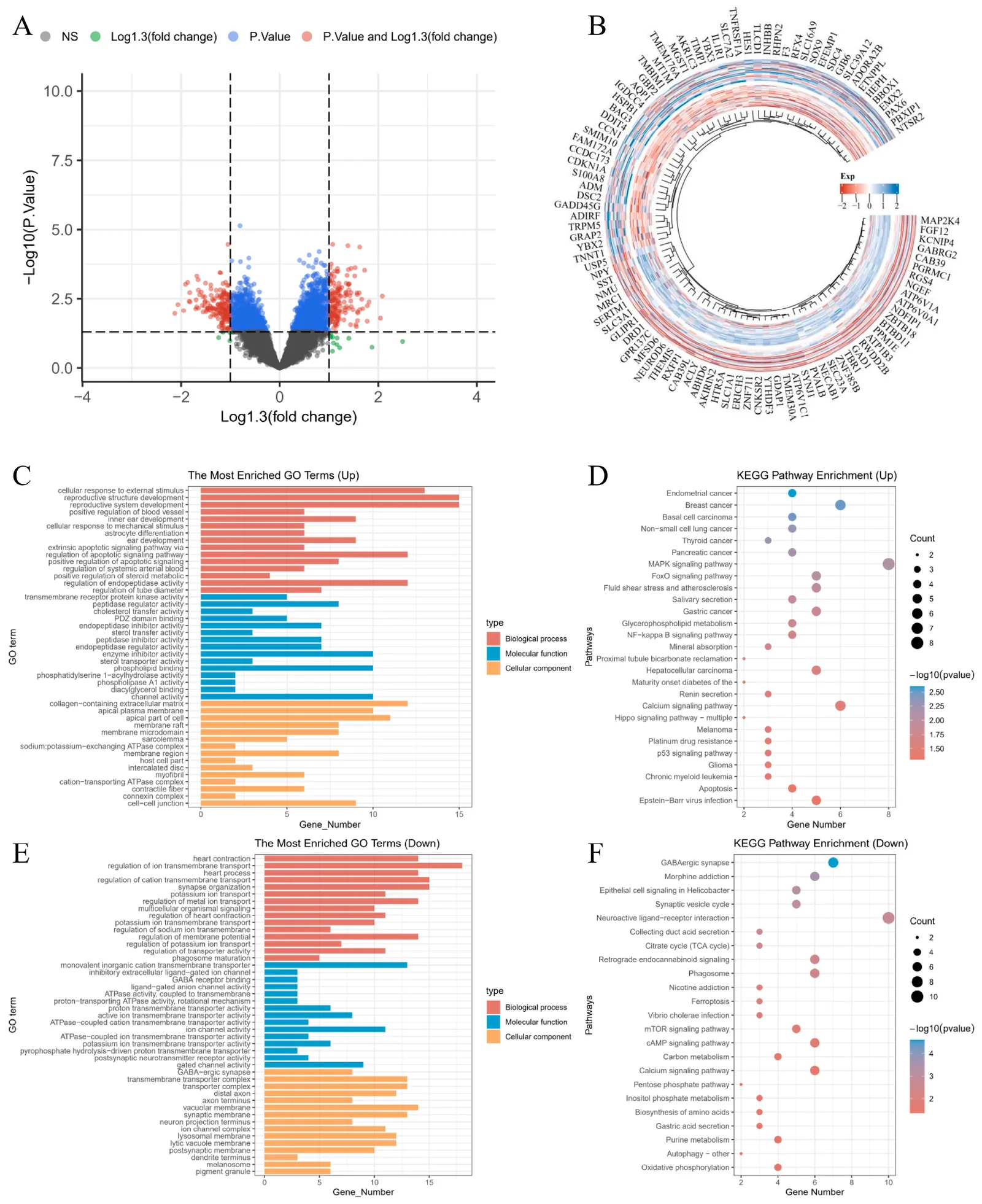
Screening and pathway enrichment analysis of SCZ-related differentially expressed genes. (**A**,**B**): Differentially expressed genes from the GSE21138 dataset. (**C**–**F**): GO and KEGG analysis of the differentially expressed genes.

As shown in Figure 2A,B, a total of 317 differentially expressed genes were identified (151 upregulated and 166 downregulated). GO and KEGG pathway enrichment analyses of these differentially expressed genes showed that the highly expressed genes were mainly involved in the cellular response to external stimulus, transmembrane receptor protein kinase activity, the collagen-containing extracellular matrix, the MAPK signaling pathway, the FoxO signaling pathway, and the NF-κB signaling pathway. The downregulated genes were mainly involved in heart contraction, the monovalent inorganic cation transmembrane transporter, the GABA-ergic synapse, and ferroptosis (Figure 2C–F).

### 3.9. Screening and Pathway Enrichment Analysis of Characteristic Genes Related to Liver Metabolism in SCZ

**Figure 3:**
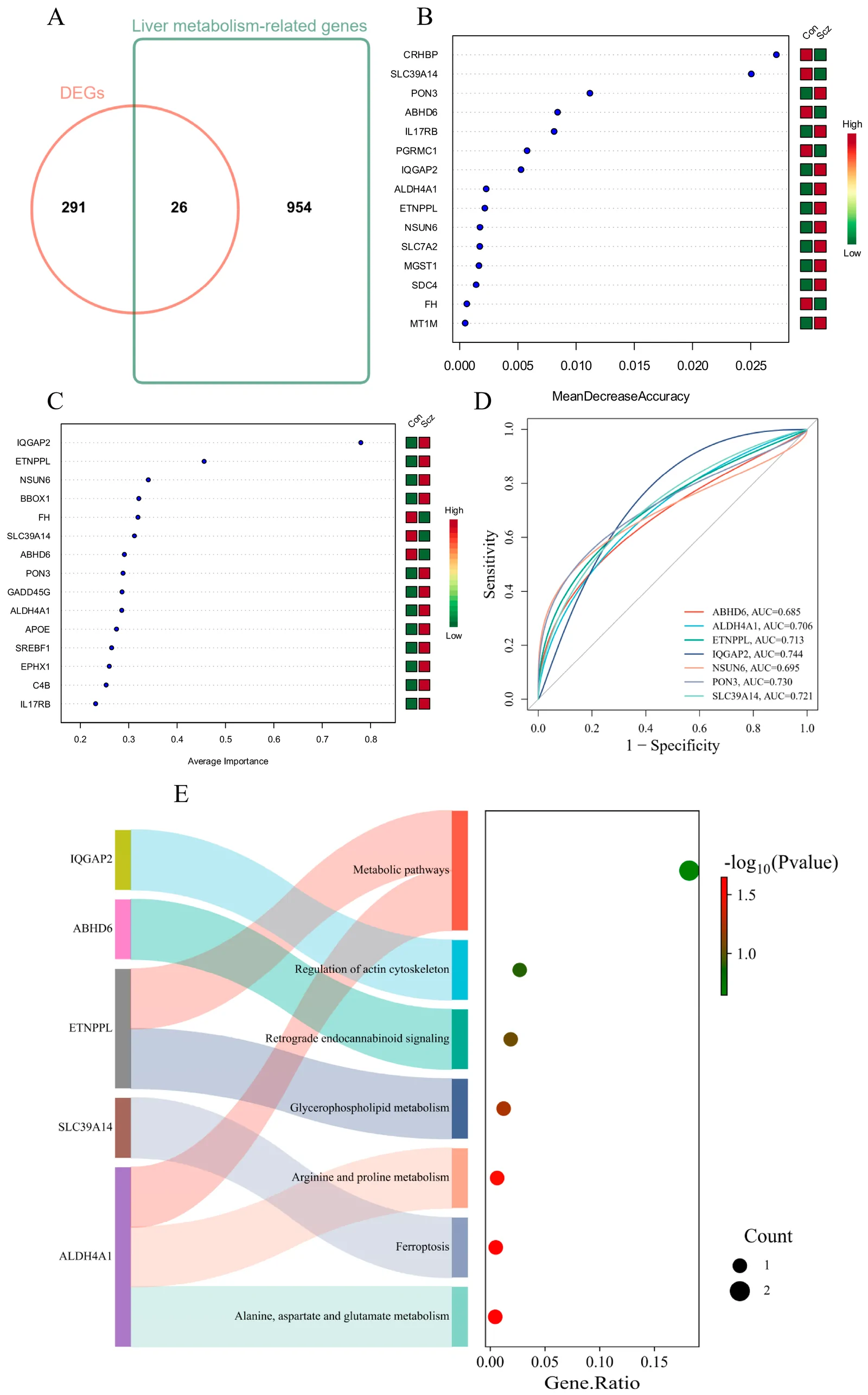
Screening and pathway enrichment analysis of liver metabolic characteristic genes in SCZ. (**A**): Intersection of SCZ-related genes and liver metabolism-related genes. (**B**,**C**): Screening of hub genes using SVM and random forest. (**D**): ROC curves for each hub gene. (**E**): KEGG pathway enrichment analysis of hub genes.

As shown in Figure 3A, there were 26 genes in common between the differentially expressed genes and liver metabolism-related genes, which we defined as characteristic genes. Further screening of these characteristic genes was performed using SVM and random forest algorithms, and the top 10 genes from each method were intersected to obtain 7 hub genes (Figure 3B,C). To evaluate the diagnostic performance of the hub genes, we used ROC curves. The results showed that the AUC of all 7 hub genes was greater than 0.6 (Figure 3D). As shown in Figure 3E, the hub genes were mainly involved in Metabolic pathways, Regulation of actin cytoskeleton, Retrograde endocannabinoid signaling, Glycerophospholipid metabolism, Arginine and proline metabolism, Ferroptosis, and Alanine, aspartate and glutamate metabolism, among others.

### 3.10. Immune Cell and Subtype Analysis

**Figure 4:**
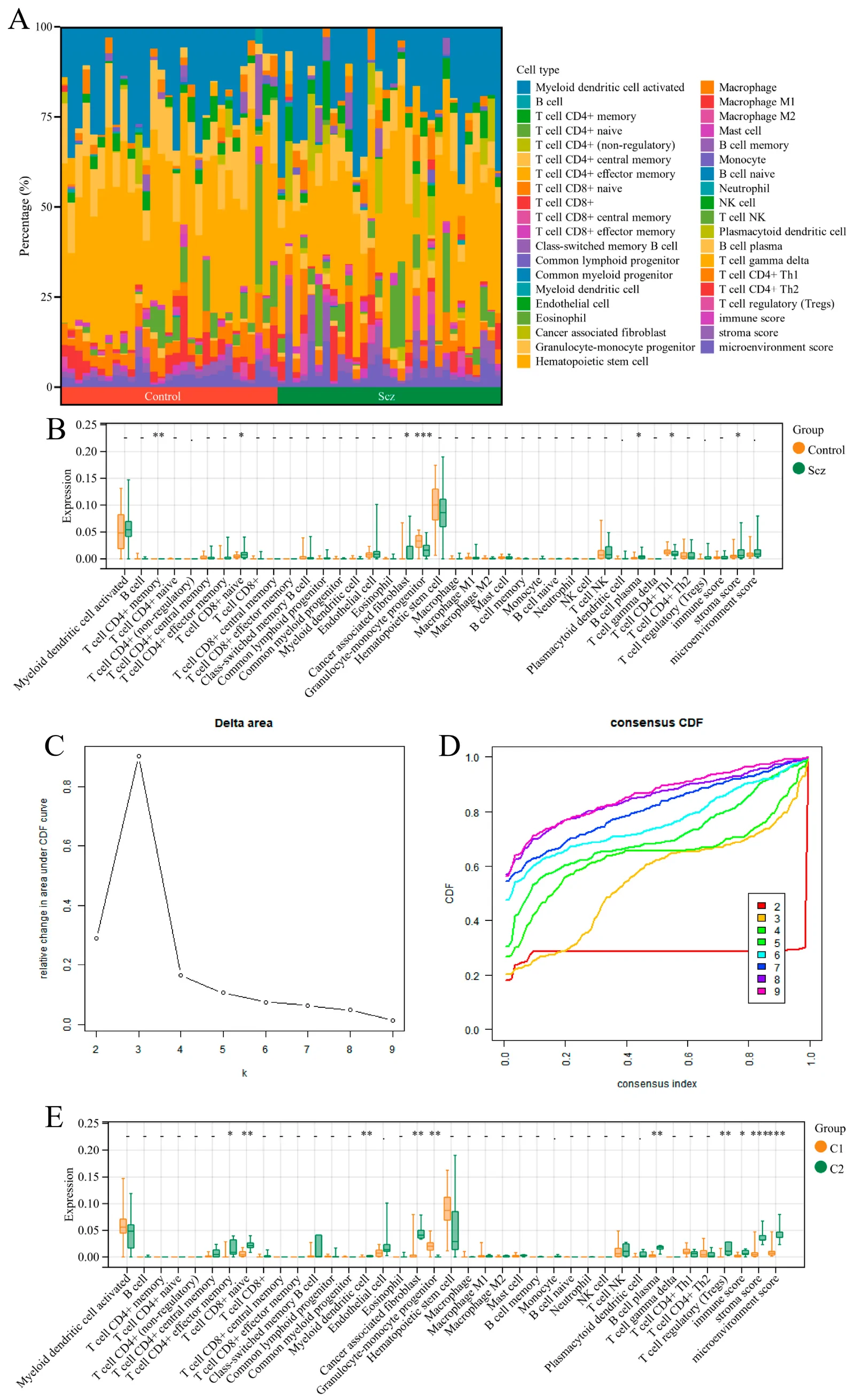
Analysis of immune cells and subtypes. (**A**): Proportions of immune cells in each group. (**B**): Comparison of immune cells between the two groups. (**C**,**D**): CDF analysis plots of subtypes based on characteristic genes. (**E**): Comparison of immune cells in subgroups. *—*p* < 0.05; **—*p* < 0.01; ***—*p* < 0.001.

As shown in Figure 4A, we used the xCell algorithm to evaluate the immune cell composition of each group. Four types of immune cells (B cell, T cell CD8^+^ naive, Cancer-associated fibroblast, and B cell plasma) were highly expressed in the SCZ group, while two types of immune cells (Granulocyte–monocyte progenitor and T cell CD4 + Th1) were highly expressed in the Control group. Furthermore, the stroma score was significantly higher in the SCZ group than in the Control group (Figure 4B). Further subtype analysis of SCZ based on hub genes showed that the best classification effect was achieved when K = 2 (Figure 4C,D). As shown in Figure 4E, compared with the C1 group, six types of immune cells (T cell CD4 + effector memory, T cell CD8^+^ naive, Myeloid dendritic cell, Cancer-associated fibroblast, B cell plasma, and T cell regulatory (Tregs)) showed significantly increased expression in the C2 group, and the immune score, stroma score, and microenvironment score were all significantly higher in the C2 group than in the C1 group. Therefore, we defined the C1 group as the low-immunity group and the C2 group as the high-immunity group.

### 3.11. Screening and Molecular Docking of Targeted Intervention Agents

**Figure 5:**
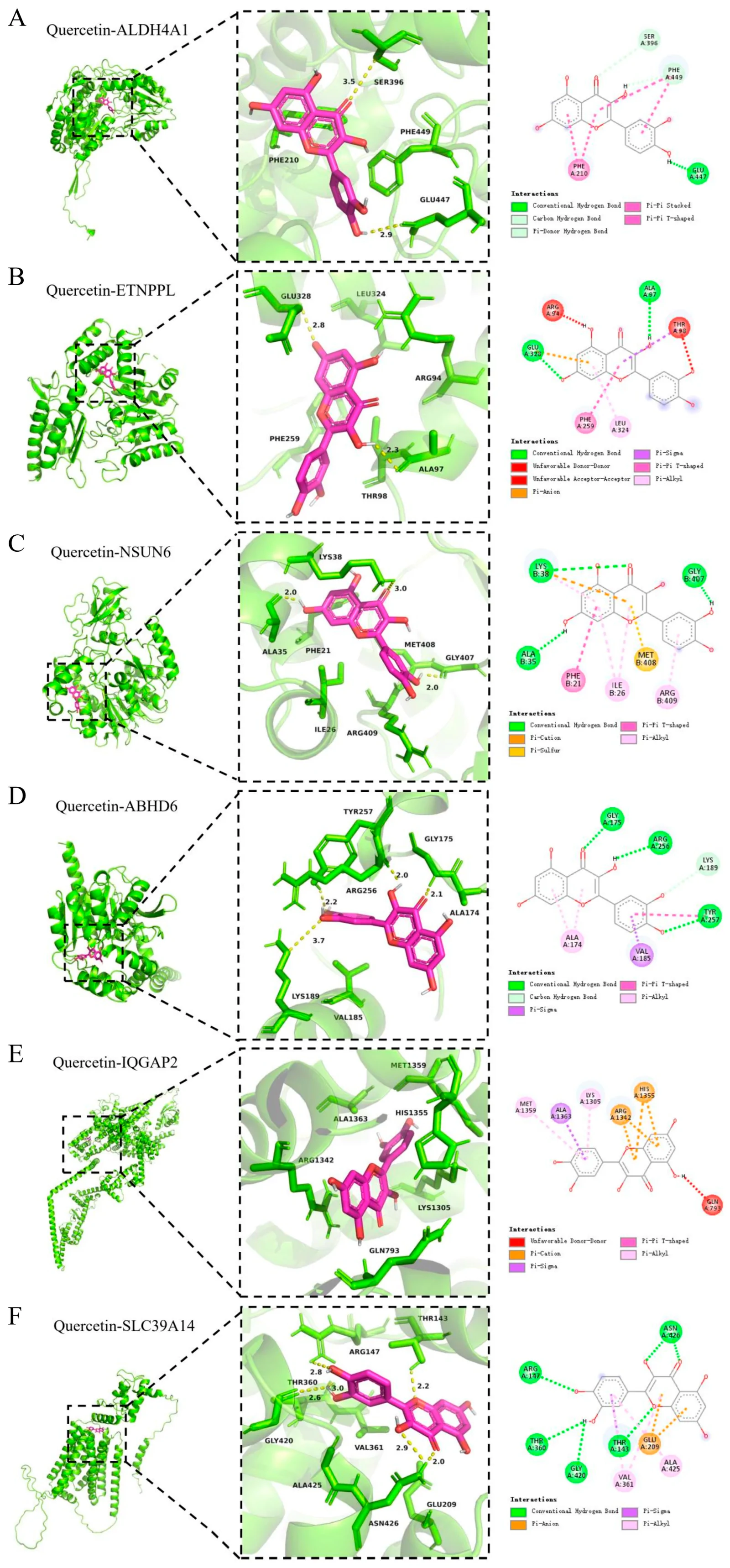
Molecular docking between quercetin and ALDH4A1 (**A**), ETNPPL (**B**), NSUN6 (**C**), ABHD6 (**D**), IQGAP2 (**E**), and SLC39A14 (**F**).

As shown in Table 7, we screened the top 10 targeted intervention agents based on hub genes. Given the advantages of natural compounds such as low toxicity and fewer side effects, quercetin was selected as a candidate intervention agent. As shown in Figure 5A–F, quercetin can bind to ALDH4A1, ETNPPL, NSUN6, ABHD6, IQGAP2, and SLC39A14 through various methods such as hydrogen bonds and π-π bonds. The binding energies were −8.068 kcal/mol, −7.007 kcal/mol, −8.117 kcal/mol, −8.319 kcal/mol, −8.467 kcal/mol, and −8.065 kcal/mol, respectively. All of the above binding energies are ≤−5.0 kcal/mol, indicating that quercetin has a strong binding ability with ALDH4A1, ETNPPL, NSUN6, ABHD6, IQGAP2, and SLC39A14.

## 4. Discussion

This study, based on a prospective cohort design, integrated clinical indicators and transcriptome data to systematically analyze the hepatic metabolic characteristics, immune cell infiltration status, and potential molecular targets in SCZ patients undergoing antipsychotic drug treatment. It also identified hub genes significantly associated with metabolic disorders. To address the inconsistency in background information between publicly available transcriptomic data and clinical cohorts, this study clearly defines their complementary relationship: the public dataset GSE21138-which comprises transcriptomic profiles of the prefrontal cortex from patients with SCZ and healthy controls-was utilized to elucidate the molecular mechanisms underlying liver-immune interactions, despite the dataset’s lack of comprehensive demographic details, records of antipsychotic medication use, and longitudinal phenotypic data. In contrast, our self-enrolled cohort comprising 340 SCZ patients with stable risperidone/chlorpromazine treatment and 340 age- and gender-matched healthy controls is equipped with unified medication regimens and rigorous two-year follow-up biochemical monitoring data, which effectively validates and supplements the medication-related phenotypic characteristics undetectable in public transcriptomic datasets, thus enhancing the clinical translational value of our molecular inferences. Subsequently, through targeted intervention agent screening and molecular docking analysis, quercetin was preliminarily identified as a novel natural compound intervention strategy for the “high-inflammation subtype” of SCZ. This series of work provides new clues for understanding the mechanisms of antipsychotic drug-induced metabolic abnormalities and developing potential intervention strategies.

### 4.1. Effects of Antipsychotic Drugs on Metabolism and Liver Function in SCZ Patients

Cross-sectional comparisons adjusted for age and gender between 340 SCZ patients and 340 matched healthy controls first revealed inherent hematological and metabolic disturbances at baseline: patients exhibited elevated inflammatory leukocytes (WBC, NEUT, MONO), reduced eosinophil proportion, anemic erythrocyte profiles (lower RBC, HGB, HCT, MCHC) and universally increased platelet biomarkers including PLT, PCT and MPV, demonstrating innate immune activation and erythroid/platelet dysregulation independent of long-term medication progression. The clinical longitudinal data from this study further showed that after two years of consistent antipsychotic therapy, SCZ patients exhibited longitudinal changes in multiple metabolic and liver function indicators, which differed from the cross-sectional between-group differences observed at baseline. Specifically, FBG decreased, and HDL increased, which is consistent with the complex effects of antipsychotic drugs on glucose and lipid metabolism observed in some studies [15,16]. However, it is worth noting that our study also found that liver function indicators such as ALT, AST, and TBIL levels were significantly reduced, while ALB, GLB, and UREA levels were significantly increased. Notably, urea exhibited divergent patterns in cross-sectional and longitudinal analyses: baseline urea concentrations were markedly lower in patients relative to healthy controls, while urea levels increased over the 2-year follow-up under prolonged antipsychotic exposure. This finding may reflect altered protein catabolism, and we note that urea alone is insufficient to confirm progressive renal impairment in the absence of additional renal function indicators. These changes may reflect adaptive adjustments in hepatic synthesis and metabolic function due to long-term drug treatment, and may also be related to the improvement in overall health status after the patient’s condition stabilized.

Regarding blood drug concentrations, we found that serum chlorpromazine concentrations significantly decreased after follow-up, while the concentration of its active metabolite, 9-OH-RISP, significantly increased. Chlorpromazine, a classic antipsychotic drug, is primarily metabolized by the liver, and its blood drug concentration varies considerably among individuals. Correlation analysis further revealed a significant negative correlation between increased 9-OH-RISP concentration and decreased ALT levels, and a significant positive correlation with increased DBIL levels. Simultaneously, increased chlorpromazine concentration was associated with increased GLB levels and decreased A/G ratio. These associations suggest that antipsychotic drug metabolites may indirectly affect the body’s immune and inflammatory state by regulating liver metabolism and synthesis functions, forming a cascade reaction of “drug metabolism–hepatic response–immune regulation”. 9-OH-RISP is the main active metabolite of risperidone, catalyzed by the CYP2D6 enzyme, and its concentration changes better reflect the drug’s active state in the body [17]. Long-term exposure to antipsychotic drugs may reduce hepatocyte stress by stabilizing blood drug concentrations, thereby reducing ALT release [18]; in addition, some drugs can inhibit the activity of bilirubin conjugating enzyme UGT1A1, leading to a slight increase in DBIL [19,20]. Bilirubin, as the end product of liver metabolism, its level changes not only reflect the fine-tuning of liver uptake, conjugation, and excretion functions, but are also closely related to inflammatory responses. Previous studies have confirmed that bilirubin has potential anti-inflammatory activity and can downregulate the expression of pro-inflammatory factors by inhibiting NF-κB pathway activation. Its increased level may be a compensatory response of the body to drug-induced mild inflammation [21,22], which aligns with our cross-sectional baseline finding of elevated circulating inflammatory cells in SCZ patients compared to healthy controls and the stratification of a high-inflammation subtype within our patient cohort, suggesting that drug metabolites may participate in inflammatory homeostasis regulation by regulating bilirubin metabolism.

GLB is mainly synthesized by the liver and secreted by plasma cells. Its increased level usually indicates immune activation or chronic inflammation, while a decreased A/G ratio is a typical manifestation of liver synthesis dysfunction and immune dysregulation [23]. Our baseline cross-sectional analysis already confirmed generalized immune hyperactivity in SCZ patients relative to 340 healthy controls, and longitudinal follow-up further illustrated that this inflammatory phenotype is amplified by sustained chlorpromazine exposure. Chlorpromazine, a classic antipsychotic drug, can induce metabolic stress in the liver with long-term use. On the one hand, it stimulates the liver to synthesize immunoglobulins, enhancing humoral immune responses; on the other hand, it may disrupt liver–immune crosstalk, promoting plasma cell activation and antibody secretion, further exacerbating the immune inflammatory response [24,25]. Combined with the results showing significantly increased plasma cell infiltration in the high-inflammation subtype mentioned earlier, this suggests that elevated chlorpromazine levels may exacerbate immune dysregulation in patients with the high-inflammation subtype by upregulating globulin levels. When benchmarked against normative data from our 340-subject healthy control group, the A/G ratio can serve as a composite indicator for assessing the degree of immune inflammation and liver function in patients, providing objective evidence for clinically identifying patients at high metabolic risk and with a high inflammatory state. Stratified regression models adjusted for age and sex verified distinct hepatic correlates for the two antipsychotics. 9-hydroxyrisperidone was associated with multiple liver function parameters in risperidone recipients, while chlorpromazine concentration mainly correlated with AST and globulin. These drug-specific effects underpin the heterogeneous metabolic and inflammatory manifestations among treated patients.

### 4.2. Identification and Cross-Tissue Functional Exploration of Core Metabolic and Inflammatory Genes in SCZ

By deeply mining transcriptome data of SCZ from the GEO database, we first screened out 317 DEGs. Functional enrichment analysis showed that these DEGs were mainly involved in cellular response to external stimuli, transmembrane receptor protein kinase activity, extracellular matrix, and signaling pathways such as MAPK, FoxO, and NF-κB. These pathways play a crucial role in regulating cell proliferation, differentiation, apoptosis, and immune inflammatory responses, and their abnormal activation has been widely confirmed to be related to the pathophysiological process of SCZ [23,26]. Given the clinical alterations in hepatic and metabolic profiles observed in SCZ patients in our cohort, we further intersected the brain-derived DEGs with liver metabolism-related gene sets from the HPA database to explore potential cross-tissue shared metabolic regulators. This screening yielded 26 candidate genes with putative metabolic functions. Subsequent machine learning validation using support vector machine (SVM) and random forest (RF) algorithms identified seven core hub genes, including *ABHD6*, *ALDH4A1*, *ETNPPL*, *IQGAP2*, *NSUN6*, *SLC39A14*, and *TRPM5*. These hub genes exhibited favorable diagnostic efficiency for distinguishing SCZ from healthy controls (AUC > 0.6) based on brain transcriptomic profiles. All gene expression analyses in the present study were derived from brain tissue transcriptomic data and do not directly represent hepatic gene expression patterns. The identified genes were explored only for cross-tissue functional relevance to liver metabolic and immune pathways, rather than defined as specific liver-derived or liver-signature genes. The potential translational link between brain transcriptomic alterations and peripheral hepatic metabolic abnormalities remains speculative and requires further tissue-specific validation. Consistent with this exploratory cross-tissue implication, the core genes identified from brain transcriptomic profiling were further supported by significant baseline differences in hepatic, lipid, and immune biomarkers between 340 SCZ patients and 340 matched healthy controls in our clinical cohort.

Notably, our study found that ALDH4A1 is abnormally expressed in SCZ patients. ALDH4A1 is a mitochondrial enzyme mainly involved in proline metabolism, and its dysfunction is associated with various neurological diseases [24]. Studies have found that ALDH4A1 expression levels are significantly increased in the post mortem brain tissue of SCZ patients and are associated with gene variations of proline metabolism-related enzymes, suggesting that it may play an important role in the pathogenesis of SCZ [25]. ABHD6 (α/β hydrolase domain-containing protein 6) is an endogenous cannabinoid hydrolase that participates in the regulation of lipid metabolism and energy homeostasis by regulating the level of 2-arachidonoylglycerol (2-AG). It is expressed in both the liver and brain and is closely related to obesity and insulin resistance [27,28]. ETNPPL (ethanolamine phosphate phosphatase-like protein) is involved in glycerophospholipid metabolism, and abnormalities in glycerophospholipid metabolism have also been reported in patients with SCZ [29]. The identification of these genes not only provides new molecular targets for understanding hepatic metabolic disorders in SCZ, consistent with the broad-spectrum lipid and hepatic biomarker disparities between our patient and healthy control cohorts, but also offers potential directions for developing novel antipsychotic drugs with hepatoprotective effects. Nevertheless, we adopted prefrontal cortex transcriptomic data from the public dataset GSE21138 for preliminary gene screening. This dataset was selected primarily to capture the intrinsic molecular features of schizophrenia, given its primary nature as a neuropsychiatric disorder. We acknowledge that brain tissue cannot directly reflect antipsychotic-associated hepatic transcriptional changes. Since high-quality public liver transcriptomic datasets for schizophrenia patients remain unavailable, we integrated longitudinal clinical hepatic function, metabolic parameters and serum drug concentration data from our prospective cohort. Such combined design enables us to dissect the hepato-immune crosstalk along the brain–liver axis, bridging central disease-related molecular alterations and peripheral drug-mediated liver metabolic abnormalities. We recognize that transcriptional profiles in brain tissue cannot completely mirror hepatocyte gene expression, which should be addressed in future investigations.

### 4.3. Subtype Analysis of SCZ Based on Hub Genes and Their Immunological Characteristics

Based on the expression profiles of seven hub genes, we classified SCZ patients into two distinct molecular subtypes (C1 and C2) via unsupervised consensus clustering. Comprehensive evaluation identified two as the optimal cluster number (K = 2), indicating detectable immune-related molecular heterogeneity among SCZ patients associated with liver metabolism-relevant genes.

From subtype-specific immune microenvironment profiling, we designated C1 as the low-immune subtype and C2 as the high-immune subtype. This subtyping aligns with our immune cell composition analyses using the xCell algorithm. The computational estimates from xCell revealed that scores for B cells, naive CD8^+^ T cells, cancer-associated fibroblasts, plasma B cells and stromal components were higher in SCZ patients relative to healthy controls, whereas granulocyte–monocyte progenitors and CD4^+^ Th1 T-cell scores were lower. These computationally derived estimates suggest altered enrichment of immune and stromal compartments as a shared immune feature in SCZ. Subtyping based on hub genes further partitions this shared feature into subtype-specific patterns, offering a framework to explore disease heterogeneity.

Specifically, we found that the infiltration levels of B cells and plasma cells were significantly increased in the peripheral blood of SCZ patients. B cells are not only the main executors of humoral immunity but also participate in immune regulation and inflammatory responses by secreting cytokines [30]. Increasing evidence suggests that SCZ patients exhibit autoantibodies and complement system activation, which may be related to abnormal B cell function [31,32]. In addition, abnormal activation of CD8^+^ T cells is also considered an important component of the immunopathology of SCZ, which may damage nerve cells through direct cytotoxic effects or by secreting pro-inflammatory cytokines [33]. From the perspective of immune regulatory mechanisms, the immune cells and stromal components enriched in the C2 subtype collectively construct a highly inflammatory microenvironment: T cell CD4 + effector memory, as an activated T cell subset that has experienced antigen stimulation, can rapidly proliferate and secrete a large number of pro-inflammatory factors (such as IFN-γ and IL-17), exacerbating the local inflammatory response [9]; the enrichment of T cell CD8^+^ naive suggests that SCZ patients may have abnormal antigen presentation, driving the activation and proliferation of naive T cells and participating in adaptive immune dysregulation [34]; myeloid dendritic cells, as key antigen-presenting cells, can use their abnormal enrichment to enhance antigen presentation efficiency and further activate T and B cell immune responses [35]; B cell plasma, as the terminal stage of B cell differentiation, can secrete a large amount of antibodies, and combined with previous studies showing abnormal autoantibodies in SCZ patients, it is speculated that they may participate in the occurrence of autoimmune reactions [36]; and although T cell regulatory (Tregs) mainly exert immunosuppressive effects, their abnormal enrichment in the highly inflammatory microenvironment may be a compensatory response of the body, and this insufficient compensation may further exacerbate immune imbalance [37]. Particularly noteworthy is the significant enrichment of cancer-associated fibroblasts (CAFs) in the C2 subtype, which for the first time reveals the crucial role of stromal cells in the highly inflammatory subtype of SCZ.

CAF are typically associated with tissue repair and fibrosis, but in the inflammatory microenvironment, they can also promote the persistence and amplification of inflammatory responses by secreting various cytokines and growth factors [38]. From a biological function perspective, CAFs are not simply “tissue repair cells”; in the inflammatory microenvironment, they can construct a sustained “inflammatory amplification loop” by secreting various soluble mediators such as IL-6, TNF-α, chemokines, and growth factors [39,40]. On the one hand, these factors can directly activate immune cells such as macrophages and T cells, promoting the release of pro-inflammatory factors and exacerbating systemic inflammatory responses [41]; on the other hand, matrix metalloproteinases (MMPs) secreted by CAFs can remodel the tissue microenvironment, disrupting intercellular homeostasis and further amplifying the vicious cycle of immune dysregulation and metabolic imbalance [42]. Notably, all CAF enrichment patterns in the present study were predicted computationally via xCell analysis and represent estimated immune-cell scores rather than direct experimental evidence of hepatic CAF infiltration. Based on the theoretical framework of liver–immune crosstalk, we herein propose a speculative mechanistic model linking predicted CAF alterations to antipsychotic-associated metabolic perturbations. Specifically, antipsychotic metabolism may trigger subtle hepatic microenvironmental changes that potentially induce CAF activation. The subsequent release of inflammatory and metabolic mediators may further propagate systemic inflammation and interfere with peripheral metabolic organs, including adipose tissue and the pancreas, thereby theoretically contributing to abnormal glucose and lipid metabolism.

### 4.4. Screening of Targeted Intervention Agents and the Therapeutic Potential of Quercetin

Among all candidate compounds screened as targeted intervention agents, Cyclosporine A achieved the highest score. However, Cyclosporine A is a potent clinical immunosuppressant associated with significant hepatotoxicity, nephrotoxicity, and excessive systemic immunosuppression [43]. Given our research objective—long-term adjunctive intervention for chronic metabolic and hepatic–immune damage induced by antipsychotic medications in patients with SCZ-the severe organ toxicity of Cyclosporine A renders it unsuitable for long-term clinical administration in this population.

Quercetin, a natural flavonoid compound, has been proven to have a wide range of pharmacological activities. Numerous studies have shown that quercetin exerts powerful anti-inflammatory effects by inhibiting inflammatory pathways such as NF-κB and MAPK, and downregulating the expression of pro-inflammatory factors such as IL-6 and TNF-α [44,45,46]. Simultaneously, quercetin can improve glucose and lipid metabolic disorders and insulin resistance through various mechanisms, including regulating insulin signaling pathways, inhibiting intestinal glucose absorption, and promoting lipolysis [47]. Furthermore, quercetin also has neuroprotective effects, improving cognitive impairment in schizophrenic model mice by activating the PI3K/AKT/CREB/BDNF signaling pathway [48].

Our molecular docking results provide an “in silico” mechanistic hypothesis for potential biological interactions of quercetin in SCZ. Quercetin may theoretically modulate hepatic metabolism and excessive immune inflammation through predicted binding to our identified hub genes, potentially disrupting the “metabolic disorder–inflammation” cycle. This is consistent with prior network pharmacology studies reporting multi-target interactions of quercetin in complex diseases such as metabolic syndrome.

### 4.5. Limitations of the Study

Although this study yielded several meaningful findings, there are some notable limitations. First, this is a single-center prospective cohort with a relatively limited sample size and no external cohort validation. Larger multi-center studies with diverse populations are required to verify our findings and improve generalizability. Second, transcriptomic data were retrieved from the public GSE21138 dataset with a small sample size, which may affect the stability of DEG and subtype analyses. In addition, this public dataset lacks detailed clinical and medication records, and brain tissue gene expression profiles cannot directly represent transcriptional patterns in hepatocytes. Future work should collect liver-derived biospecimens for further validation. Third, our interpretation of longitudinal changes relies on paired *t*-tests for within-patient comparisons, as healthy controls only had baseline measurements without follow-up data. Consequently, we could not apply repeated-measures ANOVA or linear mixed-effects models to assess group-by-time interactions between SCZ patients and controls. Fourth, this study relies heavily on bioinformatic analyses and in silico molecular docking, without validation from in vivo animal studies or clinical intervention trials. Although cyclosporine A achieved the highest score in our drug screening, only theoretical comparisons were performed, and no cellular functional assays were conducted to compare its efficacy and toxicity with quercetin. Further animal models are needed to validate the functions of these hub genes and the biological interactions of quercetin.

## 5. Conclusions

In summary, this observational study identified antipsychotic-associated alterations in hepatorenal and immune-metabolic profiles, and characterized liver-relevant hub genes for stratifying high- and low-inflammation subtypes. The multi-target flavonoid quercetin shows potential biological interactions for patients with high-inflammatory subtypes, although these in silico findings require further validation. This work provides insights into hepato-immune correlates of antipsychotic-related metabolic complications and offers preliminary clues for stratified management.

## Figures and Tables

**Table 1:** Comparison of complete blood count results of the study participants.

CBC	SCZ-A (*n* = 340)	SCZ-B (*n* = 340)	*t*/*χ*^2^/Z	*p*
WBC	6.868 ± 1.865	6.884 ± 1.831	−0.108	0.914
NEUT	4.198 ± 1.505	4.213 ± 1.470	−0.133	0.894
NEUT%	60.409 ± 9.097	60.298 ± 9.051	0.160	0.873
LYMPH	2.055 ± 0.709	2.078 ± 0.701	−0.418	0.676
LYMPH%	30.504 ± 8.383	30.726 ± 8.368	−0.344	0.731
MONO	0.456 ± 0.142	0.460 ± 0.153	−0.409	0.683
MONO%	6.762 ± 1.684	6.772 ± 1.909	−0.070	0.944
EOS	0.126 ± 0.129	0.119 ± 0.104	0.763	0.446
EOS%	1.832 ± 1.731	1.752 ± 1.500	0.643	0.251
BASO	0.034 ± 0.017	0.031 ± 0.016	2.118	0.035
BASO%	0.492 ± 0.235	0.452 ± 0.224	2.257	0.024
RBC	4.702 ± 0.507	4.695 ± 0.494	0.176	0.861
HGB	139.867 ± 16.534	140.742 ± 16.519	−0.686	0.493
MCH	29.789 ± 2.218	30.011 ± 2.220	−1.297	0.195
MCHC	328.112 ± 11.192	329.324 ± 11.360	−1.393	0.164
HCT	42.565 ± 4.343	42.674 ± 4.323	−0.327	0.744
MCV	90.711 ± 5.084	91.055 ± 5.104	−0.874	0.382
RDW-SD	42.258 ± 3.207	42.599 ± 3.173	−1.393	0.164
RDW-CV	12.762 ± 1.074	12.845 ± 1.285	−0.912	0.362
PLT	253.510 ± 73.324	239.787 ± 75.710	2.387	0.017
PCT	0.266 ± 0.072	0.255 ± 0.074	2.009	0.045
MPV	10.610 ± 0.948	10.750 ± 0.991	−1.881	0.060
PDW	0.127 ± 0.021	0.131 ± 0.023	−2.418	0.016
P-LCR	29.817 ± 7.685	31.069 ± 7.906	−2.092	0.037

Note: CBC: Complete Blood Count. WBC: White Blood Cell Count. NEUT: Neutrophil Count. NEUT%: Neutrophil Percentage. LYMPH: Lymphocyte Count. LYMPH%: Lymphocyte Percentage. MONO: Monocyte Count. MONO%: Monocyte Percentage. EOS: Eosinophil Count. EOS%: Eosinophil Percentage. BASO: Basophil Count. BASO%: Basophil Percentage. RBC: Red Blood Cell Count. HGB: Hemoglobin. MCH: Mean Corpuscular Hemoglobin. MCHC: Mean Corpuscular Hemoglobin Concentration. HCT: Hematocrit. MCV: Mean Corpuscular Volume. RDW-SD: Red Cell Distribution Width-SD. RDW-CV: Red Cell Distribution Width-CV. PLT: Platelet Count. PCT: Plateletcrit. MPV: Mean Platelet Volume. PDW: Platelet Distribution Width. P-LCR: Platelet Large Cell Ratio. SCZ-A: Baseline data for patients with SCZ. SCZ-B: Data obtained from SCZ patients after a two-year follow-up.

**Table 2:** Comparison of two sets of liver and kidney function indicators.

LFTs	SCZ-A (*n* = 340)	SCZ-B (*n* = 340)	*t*/*χ*^2^/Z	*p*
ALT	28.059 ± 18.721	24.005 ± 12.895	3.277	0.001
AST	25.434 ± 10.912	21.853 ± 7.738	4.919	<0.001
ALP	90.078 ± 25.005	90.363 ± 25.098	−0.148	0.883
GGT	32.286 ± 26.293	28.507 ± 23.534	1.965	0.05
TBIL	13.461 ± 4.998	11.792 ± 4.235	4.675	<0.001
DBIL	3.919 ± 1.803	5.000 ± 1.527	−8.403	<0.001
TP	69.514 ± 3.677	71.104 ± 4.019	5.357	<0.001
ALB	44.625 ± 2.108	45.032 ± 2.853	−2.102	0.036
GLB	24.889 ± 3.418	26.072 ± 3.385	−4.511	<0.001
A/G	1.828 ± 0.295	1.754 ± 0.269	3.447	0.001
TBA	3.214 ± 2.731	2.738 ± 2.559	2.332	0.020
UREA	4.780 ± 1.390	5.155 ± 1.576	−3.276	0.001
UA	359.789 ± 92.119	316.476 ± 76.170	6.657	<0.001

Note: LFTs: Liver Function Tests. TBIL: Total Bilirubin. DBIL: Direct Bilirubin. TP: Total Protein. ALB: Albumin. GLB: Globulin. A/G: Albumin/Globulin Ratio. TBA: Total Bile Acids. UREA: Urea. UA: Uric Acid.

**Table 3:** Comparison of blood lipid and blood glucose levels between two groups.

Blood Lipids and Glucose	SCZ-A (*n* = 340)	SCZ-B (*n* = 340)	*t*/*χ*^2^/Z	*p*
CHOL	5.106 ± 1.016	5.012 ± 1.005	1.208	0.227
TG	2.058 ± 1.682	1.905 ± 1.478	1.253	0.211
HDL	1.267 ± 0.291	1.341 ± 0.326	−3.114	0.002
LDL	3.049 ± 0.804	2.993 ± 0.820	0.893	0.372
FBG	15.662 ± 6.354	6.597 ± 2.469	3.187	0.033

Note: CHOL: Total Cholesterol. FBG: Fasting Blood Glucose.

**Table 4:** Comparison of blood drug concentrations between two groups.

TDM	SCZ-A (*n* = 340)	SCZ-B (*n* = 340)	*t*/*χ*^2^/Z	*p*
RISP	12.01 ± 8.838	9.89 ± 13.315	0.695	0.489
9-OH-RISP	15.24 ± 11.757	22.67 ± 17.750	−2.634	0.010
MR	3.31 ± 23.699	0.55 ± 4.518	2.106	0.036
QTP	107.47 ± 128.214	117.75 ± 66.649	−0.289	0.774
OLZ	40.00	46.60 ± 32.466	−0.194	0.851
CPZ	149.85 ± 114.928	81.84 ± 88.808	2.786	0.007
CLZ	222.46 ± 192.193	202.77 ± 154.267	0.794	0.428
NDMC	139.75 ± 116.744	117.19 ± 85.433	1.543	0.124
ARI	122.62 ± 78.475	138.08 ± 110.462	−0.448	0.657
VPA	17.100 ± 10.579	40.267 ± 30.115	−1.271	0.233

Note: TDM: Therapeutic Drug Monitoring. RISP: Serum Risperidone Concentration. 9-OH-RISP: 9-Hydroxyrisperidone. MR: Metabolic Ratio (9-OH-Risperidone/Risperidone). QTP: Serum Quetiapine Concentration. OLZ: Serum Olanzapine Concentration. CPZ: Serum Chlorpromazine Concentration. CLZ: Serum Clozapine Concentration. NDMC: N-Desmethylclozapine (Norclozapine). ARI: Serum Aripiprazole Concentration. VPA: Serum Valproate Concentration.

**Table 5:** Multivariate linear regression analysis for associations between biochemical indices and drug concentrations.

Variable	B	SE	β	*t*	*p*
BASO	−0.003	0.001	−0.079	−2.101	0.036
BASO%	−0.040	0.017	−0.086	−2.283	0.023
PDW	0.004	0.002	0.087	2.263	0.024
ALT	−3.440	1.188	−0.106	−2.895	0.004
AST	−3.483	0.731	−0.181	−4.764	<0.001
TBIL	−1.757	0.356	−0.187	−4.935	<0.001
DBIL	1.041	0.128	0.297	8.140	<0.001
TP	1.699	0.295	0.216	5.764	<0.001
ALB	0.525	0.180	0.105	2.912	0.004
GLB	1.174	0.258	0.170	4.544	<0.001
A/G	−0.069	0.021	−0.121	−3.296	0.001
TBA	−0.502	0.205	−0.095	−2.453	0.014
UREA	0.274	0.107	0.092	2.565	0.011
UA	−42.172	6.088	−0.242	−6.927	<0.001
HDL	0.076	0.022	0.122	3.420	0.001
FBG	−9.056	1.143	−0.392	−7.923	<0.001
9-OH-RISP	7.615	2.940	0.243	2.591	0.011
CPZ	−70.741	24.455	−0.333	−2.893	0.005

**Table 6:** Stratified Regression Analysis of Drug-Specific Hepatic Biochemical Associations.

Variable	Group	R^2^	Adjust R^2^	F	*p*
AST	9-OH-RISP	0.179	0.135	4.010	0.050
	CPZ	0.299	0.235	9.340	0.004
ALT	9-OH-RISP	0.288	0.249	4.737	0.034
	CPZ	0.146	0.068	1.225	0.276
TBIL	9-OH-RISP	0.165	0.119	5.472	0.023
	CPZ	0.104	0.023	2.650	0.113
DBIL	9-OH-RISP	0.132	0.084	3.762	0.058
	CPZ	0.045	−0.042	0.423	0.520
TP	9-OH-RISP	0.138	0.091	4.424	0.040
	CPZ	0.098	0.016	2.804	0.103
ALB	9-OH-RISP	0.125	0.077	2.268	0.138
	CPZ	0.191	0.118	0.011	0.916
GLB	9-OH-RISP	0.096	0.047	1.105	0.298
	CPZ	0.284	0.219	5.484	0.025
A/G	9-OH-RISP	0.065	0.015	0.092	0.763
	CPZ	0.318	0.257	2.825	0.102
TBA	9-OH-RISP	0.058	0.007	0.524	0.472
	CPZ	0.059	−0.026	1.209	0.279

Note: Stratified univariate general linear models were performed separately in risperidone and chlorpromazine subgroups. All models were adjusted for age and sex.

**Table 7:** Top 10 Targeted Intervention Agents.

Term	*p*-Value	Adjusted *p*-Value	Odds Ratio	Combined Score
cyclosporin A	4.740 × 10^−5^	6 × 10^−3^	106,225	1,057,661.316
quercetin	9.345 × 10^−5^	6 × 10^−3^	32.058	297.434
8-Bromo-cAMP	1 × 10^−3^	5.1 × 10^−2^	22.354	152.407
Periodate-oxidized adenosine	2 × 10^−3^	8.2 × 10^−2^	36.970	223.752
TPEN	3 × 10^−3^	8.9 × 10^−2^	31.461	180.636
Malondialdehyde	5 × 10^−3^	1.08 × 10^−1^	256.154	1362.862
acetaminophen	5 × 10^−3^	1.08 × 10^−1^	9.602	50.078
cinchocaine	8 × 10^−3^	1.33 × 10^−1^	158.508	771.886
Pirinixic acid	1 × 10^−2^	1.33 × 10^−1^	9.968	46.198
CARBON TETRACHLORIDE	1 × 10^−2^	1.33 × 10^−1^	118.839	546.006

## Data Availability

The datasets used and analyzed during the current study are available from the corresponding author on reasonable request. The data are not publicly available due to privacy.

## Supplementary Materials

The following supporting information can be downloaded at https://www.mdpi.com/article/10.3390/metabo16100733/s1, Table S1: Comparison of complete blood count results of the study participants; Table S2: Comparison of Liver and Kidney Function Indicators of the Study Subjects; Table S3: Comparison of Blood Lipid and Blood Glucose Levels of the Study Subjects; Table S4: Multivariate Linear Regression Analysis of Blood and Biochemical Markers Associated with SCZ.
