# Supplementary material for: Hepato-Immune Crosstalk Underlies Antipsychotic-Associated Metabolic Dysregulation in Schizophrenia: Quercetin as a Therapeutic Candidate for High-Inflammatory Subtypes
Source: Metabolites. 2026 Sep 29;16(10):733. doi: 10.3390/metabo16100733 (PMC13706789; doi:10.3390/metabo16100733)
Supplement: Supplementary file 1 [file metabolites-16-00733-s001.zip › metabolites-4394917-supplementary.pdf]

Table S1. Comparison of complet blood count results of the study participants.

CBC	Control (*n*=340)	SCZ-A (*n*=340)	t/$\chi 2$ /Z	*p*
WBC	6.070±1.539	6.868±1.865	-5.956	**<0.001**
NEUT	3.513±1.158	4.198±1.505	-6.493	**<0.001**
NEUT%	57.271±7.505	60.409±9.097	298.252	0.420
LYMPH	1.990±0.561	2.055±0.709	-0.820	0.412
LYMPH%	33.329±7.175	30.504±8.383	255.412	0.796
MONO	0.401±0.132	0.456±0.142	-5.244	**<0.001**
MONO%	6.665±1.564	6.762±1.684	83.959	0.542
EOS	0.135±0.113	0.126±0.129	-2.119	**0.034**
EOS%	2.215±1.703	1.832±1.731	113.718	**0.003**
BASO	0.031±0.018	0.034±0.017	2.487	0.062
BASO%	0.521±0.272	0.492±0.235	40.543	**0.002**
RBC	4.827±0.480	4.702±0.507	-3.445	**0.001**
HGB	145.165±13.994	139.867±16.534	-4.685	**<0.001**
MCH	30.126±1.868	29.789±2.218	-2.380	**0.017**
MCHC	333.038±6.880	328.112±11.192	-7.223	**<0.001**
HCT	43.571±4.024	42.565±4.343	-3.317	**0.001**
MCV	90.450±4.984	90.711±5.084	-1.275	0.202
RDW-SD	44.691±6.173	42.258±3.207	-6.546	**<0.001**
RDW-CV	13.224±1.063	12.762±1.074	-8.960	**<0.001**
PLT	211.965±54.446	253.510±73.324	-7.937	**<0.001**
PCT	0.200±0.044	0.266±0.072	-12.927	**<0.001**
MPV	9.625±1.221	10.610±0.948	-11.590	**<0.001**

*: CBC: Complete Blood Count. WBC: White Blood Cell Count. NEUT: Neutrophil Count. NEUT%: Neutrophil Percentage. LYMPH: Lymphocyte Count. LYMPH%: Lymphocyte Percentage. MONO: Monocyte Count. MONO%: Monocyte Percentage. EOS: Eosinophil Count. EOS%: Eosinophil Percentage. BASO: Basophil Count. BASO%: Basophil Percentage. RBC: Red Blood Cell Count. HGB: Hemoglobin. MCH: Mean Corpuscular Hemoglobin. MCHC: Mean Corpuscular Hemoglobin Concentration. HCT: Hematocrit. MCV: Mean Corpuscular Volume. RDW-SD: Red Cell Distribution Width-SD. RDW-CV: Red Cell Distribution Width-CV. PLT: Platelet Count. PCT: Plateletcrit. MPV: Mean Platelet Volume. SCZ-A: Baseline data for patients with schizophrenia.

Table S2. Comparison of Liver and Kidney Function Indicators of the Study Subjects.

**LFTs**	**Control (*n*=340)**	**SCZ-A (*n*=340)**	**Z**	***p***
ALB	30.507±135.594	44.625±2.108	-18.942	**<0.001**
UREA	5.092±1.265	4.780±1.390	-3.519	**<0.001**

*: LFTs: Liver Function Tests. ALB: Albumin. UA: Uric Acid.

Table S3. Comparison of Blood Lipid and Blood Glucose Levels of the Study Subjects.

**Blood Lipids and Glucose**	**Control (*n*=340)**	**SCZ-A (*n*=340)**	**t/$\chi 2$ /Z**	***p***
CHOL	4.578±0.850	5.106±1.016	-7.405	**<0.001**
TG	1.954±1.807	2.058±1.682	-3.147	**0.002**
HDL	1.200±0.300	1.267±0.291	-2.931	**0.003**
LDL	2.969±0.812	3.049±0.804	-1.496	0.135

*: CHOL: Total Cholesterol. TG: Triglycerides.

Table S4. Multivariate Linear Regression Analysis of Blood and Biochemical Markers Associated with SCZ.

Variable	B	SE	β	t	*p*
WBC	0.766	0.129	0.218	5.937	**<0.001**
NEUT	0.670	0.102	0.242	6.553	**<0.001**
MONO	0.054	0.010	0.194	5.274	**<0.001**
EOS%	-0.380	0.130	-0.110	-2.914	0.004
RBC	-0.142	0.033	-0.142	-4.322	**<0.001**
HGB	-5.483	0.948	-0.177	-5.786	**<0.001**
MCHC	-4.941	0.695	-0.257	-7.114	**<0.001**
HCT	-1.058	0.260	-0.126	-4.065	**<0.001**
RDW-SD	-2.424	0.368	-0.239	-6.590	**<0.001**
RDW-CV	-0.452	0.082	-0.207	-5.498	**<0.001**
PLT	39.456	4.851	0.291	8.134	**<0.001**
PCT	0.064	0.004	0.470	14.429	**<0.001**
MPV	0.984	0.084	0.411	11.727	**<0.001**
ALB	14.570	7.372	0.076	1.976	0.049
UREA	-0.241	0.097	-0.090	-2.473	0.014
CHOL	0.530	0.072	0.273	7.343	**<0.001**
HDL	0.069	0.022	0.116	3.101	0.002
